# Molecular Characterization of Adipose Tissue in the African Elephant *(Loxodonta africana)*

**DOI:** 10.1371/journal.pone.0091717

**Published:** 2014-03-14

**Authors:** Emeli M. Nilsson, Hernan P. Fainberg, Siew S. Choong, Thomas C. Giles, James Sells, Sean May, Fiona J. Stansfield, William R. Allen, Richard D. Emes, Alison Mostyn, Nigel P. Mongan, Lisa Yon

**Affiliations:** 1 Faculty of Medicine and Health Sciences, School of Veterinary Medicine and Science, Sutton Bonington Campus, University of Nottingham, Sutton Bonington, United Kingdom; 2 Advanced Data Analysis Centre, University of Nottingham, Sutton Bonington, United Kingdom; 3 The Nottingham Arabidopsis Stock Centre, Division of Plant Sciences, University of Nottingham, Sutton Bonington, United Kingdom; 4 The Elephant Research and Conservation Unit, Savé Valley Conservancy, Harare, Zimbabwe; 5 The Paul Mellon Laboratory, “Brunswick,” Woodditton Road, Newmarket, Suffolk, United Kingdom; Clermont Université, France

## Abstract

Adipose tissue (AT) is a dynamic and flexible organ with regulatory roles in physiological functions including metabolism, reproduction and inflammation; secreted adipokines, including leptin, and fatty acids facilitate many of these roles. The African elephant (*Loxodonta africana*) is experiencing serious challenges to optimal reproduction in captivity. The physiological and molecular basis of this impaired fertility remains unknown. AT production of leptin is a crucial molecular link between nutritional status, adiposity and fertility in many species. We propose that leptin has a similar function in the African elephant. African elephant visceral and subcutaneous adipose tissue (AT) was obtained from both sexes and a range of ages including females with known pregnancy status. RNA was extracted and histological sections created and analyzed by microarray, PCR and immunohistochemistry respectively. Gas-chromatography was used to determine the fatty acid composition of AT. Microarray expression profiling was used to compare gene expression profiles of AT from pre-pubertal versus reproductively competent adult African elephants. This study demonstrates, for the first time, *leptin* mRNA and protein expression in African elephant AT. The derived protein sequence of the elephant leptin protein was exploited to determine its relationship within the class I helical cytokine superfamily, which indicates that elephant leptin is most closely related to the leptin orthologs of *Oryctolagus cuniculus* (European rabbit), *Lepus oiostolus* (woolly hare), and members of the *Ochotonidae* (Pika). Immunohistological analysis identified considerable leptin staining within the cytoplasm of adipocytes. Significant differences in fatty acid profiles between pregnant and non-pregnant animals were revealed, most notably a reduction in both linoleic and α linoleic acid in pregnant animals. This report forms the basis for future studies to address the effect of nutrient composition and body condition on reproduction in captive and wild elephants.

## Introduction

The importance of AT as a dynamic endocrine organ has emerged in the last 15 years [1]–[3] and several adipokines, including leptin (LEP), adiponectin (ADIPOQ) and TNFα have been recognized, with wide ranging postulated actions in metabolism, inflammation and reproduction [4]–[7]. AT comprises from 1% (piglet) to 65% (elephant seal pup) of the body weight of mammalian species and has a range of roles throughout the life cycle. Brown AT in the neonatal period actively produces heat through non-shivering thermogenesis, while white AT (WAT) (the predominant AT type in adult mammals) acts as a nutrient sensor, a reservoir for excess energy to be stored as lipid, and as an active secretor of AT-derived adipokines, cytokines and fatty acids (reviewed in [8]). Crucially, AT can expand and contract during periods of nutritional excess and famine to provide a buffer for excess energy [9]. The first adipokine to be identified was leptin [10]. Plasma levels of leptin increase and decline with weight gain and loss, respectively, thus signaling changes in AT energy stores. Leptin signals satiety via the leptin receptor (LEP-R), predominantly located in the appetite regulation zones of the hypothalamus.

Leptin has been linked with reproductive activity both in terms of onset of puberty and in maintenance of reproductive function [11]. For example, intracerebroventricular leptin administration increases luteinizing hormone levels in the rat. In the absence of leptin signaling, reproductive function is poor or non-existent as evidenced in leptin deficient animal models and humans [12], [13]. In humans and many other species, a sufficient level of AT is required in the body for normal functioning of the HPG axis and leptin has been identified as a key facilitator of this and acts to convey the signal of sufficient adiposity to the HPG axis.

Fatty acids not only provide building blocks for long term storage of energy in AT and a structural basis for cell membranes, but also have crucial signaling roles, particularly in energy sensing, inflammation and reproduction. In the short term, free fatty acids act to maintain normal pancreatic β-cell function, specifically by facilitating glucose-stimulated-insulin-release. However, long term exposure of the pancreas to fatty acids leads to increased basal insulin release and inhibition of glucose-stimulated-insulin-release (reviewed in [14]). The fatty acid arachidonic acid is the precursor of prostaglandins which regulate many normal physiological processes, as well as inflammatory responses including recovery; excess adipose tissue is associated with an enhanced chronic inflammatory state which may contribute to the pathophysiology associated with obesity. Essential fatty acids (EFAs), such as linoleic acid and docosahexanoic acid (DHA), have crucial roles in fertility, establishment of pregnancy and normal fetal development [15]–[18]. These EFA must be obtained through dietary sources as mammals are incapable of *de novo* generation.

The population of captive elephants, both Asian (*Elephas maximus*) and African (*Loxodonta africana*) in Europe and North America is not self-sustaining, largely due to poor fertility, resulting in a low production of offspring. Many females have irregular estrous cycles, or have no cycles at all; in North American zoos, 10.9% of Asian elephants and 31.2% of African elephants are not cycling [19]. It is acknowledged that if a solution cannot quickly be found for these reproductive difficulties, captive elephants will face demographic extinction in North American zoos within the next 50 years [20]–[22]. Leptin may play an important role in signaling to the elephant's HPG axis that a sufficient plane of nutrition has been reached in order for key events related to reproduction to occur, and that this plane of nutrition is being maintained. Given the links between nutritional state and reproductive activities in elephants [23]–[26]; and the established role of leptin in connecting the two in many other species, it is logical to expect that leptin is important in linking these two systems in the elephant. As there are key relationships between nutrition, AT, fatty acids, adipokines and reproductive function, a better understanding of these links would enable better management of nutrition and fertility in elephant populations.

The overarching hypothesis of this work is that the fatty acid and gene expression profile of African elephant AT is comparable to that seen in other mammalian species and exhibits differences based on age, sex and reproductive status. We additionally hypothesize that leptin will be expressed in African elephant AT. Therefore this study forms the essential basis for future research to determine the relationship between diet and reproductive efficiency in captive elephant populations.

## Materials and Methods

### Ethics statement

The project and acquisition of samples was approved by the local ethics committee of the School of Veterinary Medicine and Science, University of Nottingham. Elephant samples were obtained from management-organized culling operations in Save Valley Conservancy (SVC) in Zimbabwe during 2009–2011. The Zimbabwe Parks and Wildlife Management Authority (PWMA) gave permits to SVC to cull the animals and SVC gave the authors permission to use the samples. Sample collections were opportunistic, no animals were killed specifically for this study, and all permission was obtained from the relevant authorities.

### African elephant adipose tissue samples

Visceral and subcutaneous adipose tissue and adrenal glands were collected from wild African elephants (N = 23) in Zimbabwe (approx. −20 latitude, 32 longitude). The profiles of the animals are outlined in Table 1. Samples approximately 4×4 cm were collected from each animal, and rapidly divided. Half of each sample was stored in liquid nitrogen and shipped to the UK prior to long term storage at −80°C. The remaining sample was immediately preserved and shipped in 10% neutral buffered formalin.

**Table 1:** Differentially expressed genes identified in male versus female elephant adipose tissues.

Affymetrix ID	Fold Change	Regulation	Official Symbol	Function
226064_PM_s_at	4.157755	up	DGAT2	Lipid homeostasis & metabolism
201539_PM_s_at	2.972423	down	FHL1	Regulation of cell growth and organ morphogenesis
210298_PM_x_at	2.722048	down	FHL1	ATP binding
214505_PM_s_at	2.273939	down	FHL1	Transcriptional repressor
202274_PM_at	2.860813	down	ACTG2	Transcription factor
234351_PM_x_at	2.715858	down	TRPS1	ATP synthesis
209120_PM_at	2.435109	down	NR2F2	ATP synthesis
208746_PM_x_at	2.340666	up	ATP5L	Transcription factor & cytoskeleton organization
210453_PM_x_at	2.242322	up	ATP5L	RNA binding protein
226137_PM_at	2.328195	down	ZFHX3	Protein binding
209488_PM_s_at	2.315450	down	RBPMS	Transcription factor & cytoskeleton organization
225852_PM_at	2.291593	down	ANKRD17	Signal transduction
242738_PM_s_at	2.272200	down	ZFHX3	Translation regulator
233955_PM_x_at	2.123121	down	CXXC5	Protein tyrosine phosphatase activity
1558142_PM_at	2.105890	down	TNRC6B	Immune response and protein folding
216988_PM_s_at	2.102763	down	PTP4A2	Regulation of translation
200807_PM_s_at	2.098166	up	HSPD1	Microtubule-based process
226939_PM_at	2.096528	down	CPEB2	Transcription regulator
209026_PM_x_at	2.094485	up	TUBB	Cellular protein metabolic process
211714_PM_x_at	2.037895	up	TUBB	Collagen biosynthetic process
201694_PM_s_at	2.075279	up	EGR1	RNA splicing and embryo development
AFFXHSAC07/X00351_5_at	2.071605	down	ACTB	Signal transduction
215076_PM_s_at	2.062285	up	COL3A1	Regulation of transcription

### Affymetrix oligonucleotide microarray and analysis

For Affymetrix (HG133+PM) oligonucleotide microarray analysis, total RNA was extracted from 100 µg of perirenal AT (n = 4) using an RNeasy Lipid Tissue Mini kit (Qiagen, Crawley West Sussex, UK). RNA purity and concentration were determined utilizing a NanoDrop ND-1000 Spectrophotometer (Labtech International Ltd., East Sussex, UK). Sample integrities were assessed using an Agilent 2100 Bioanalyzer (Agilent Technologies UK Ltd., Stockport, UK). Three independent RNA extractions were carried out from each AT sample from each of the following groups: (i) young female (3 years old), (ii) old female (32 years old), (iii) young male (3.5 years old), and (iv) old male (16 years old). The raw data was imported into GeneSpring GX12.5 and normalized using RMA (values were transformed to median baseline). Samples were filtered based on the expression of probe-sets in the raw data. Probe-sets in the lowest 20th percentile of the data were removed (and classed as background noise). After filtering, 27542 probe-sets remained. Clustering (Euclidean with Ward's linkage) and PCA were used to compare the variance between individual samples. Moderate-T Test Volcano plots with a P value threshold of 0.05 and a fold change (FC) cut-off of 2-fold were used to identify genes significantly differentially expressed between: (A) old vs. young, and (B) female vs. male. The microarray data was deposited in ArrayExpress under the accession number E-MTAB-2024 and is available without restriction.

### Identification of the putative elephant leptin gene, array validation by quantitative PCR and gene expression analysis

The BLAT tool was used to search the African elephant genome (genome build: Broad/loxAfr3) with the human *leptin* mRNA reference sequence (Genbank accession number: NM_000230.2). The identified region of sequence similarity between human *leptin* and the elephant genome was extracted, the amino acid sequence of putative elephant leptin derived, and intron-exon boundaries identified. Similarly, the putative elephant *actin* gene was identified using the human *β-actin* reference sequence (NM_001101). Forward and reverse oligonucleotide primers for PCR for elephant *leptin* (F: TCACCAGGATCAGTGACATTTCACAC; R: CAAGGTCTCCAGGCCACTGGCC) and *actin* (F: CCTGAGCGCAAGTACTCAGTGTGG; R: TTTTGTCAAGAAAAGGTGTAACGC were designed to anneal in two exons to preclude amplification of genomic DNA and to confirm the predicted intron-exon boundaries identified. Primers for qPCR were similarly designed for *DGAT* (F: GACACACAACCTGCTGACCACCAG, R: TTCTTGCTCACTTCTGTGGCCTCTGTGC) and for *FHL1* (F: CCTTTGTGGCCAAGGACAACAAGATC, R: CCAGACGGTCCCCTTGTACTCCAC). Primer sequences were checked using *in silico* PCR. The derived amino acid sequence of elephant leptin was aligned with the human leptin protein reference sequence (NP_000221) using ClustalW (PMID: 7984417).

RNA was extracted from visceral (n = 12 female, 8 male) and subcutaneous (n = 14 female, 9 male) AT using an RNeasy Plus kit (Qiagen, Crawley, UK) following the supplier's instructions. RNA quality and concentration were determined utilizing a NanoDrop ND-1000 Spectrophotometer (LabTech International, East Sussex, UK). cDNA synthesis was carried out using the Transcriptor First Strand cDNA Synthesis kit (Roche, Burgess Hill, UK) according to the manufacturer's instructions using 0.5 µg total RNA from each sample. PCR was conducted using a Techne TC-512 Thermocycler (Techne, Cambridge, UK) under the following conditions: initial denaturation at 94 °C for 5 minutes followed by 40 cycles of 94°C for 30 seconds, annealing for 30 seconds, 72°C for 30 seconds and a final extension at 72°C for 5 minutes. The annealing temperatures for *leptin* and *actin* were 62°C and 58°C respectively. For quantitative PCR, Roche SYBR green reagent and standard cycling and plate read conditions were used in a Roche Lightcycler II real time PCR system. The identity of all PCR products was confirmed by direct Sanger sequencing (Source Bioscience, Nottingham, UK).

#### Determination of tissue phospholipid composition

Adipose tissue lipid content was extracted from visceral (n = 10 female, 5 male) and subcutaneous (n = 9 female, 4 male) tissues using the Folch method [27]; the chloroform phase was then subjected to gas-chromatography (GC) to determine the fatty acid composition within phospholipids as previously described [28]. The chloroform phase was evaporated by applying a nitrogen stream and then 2 ml of hexane was used to re-dissolve each sample. Samples were trans-esterified by the method of Christie [29] and modified by Chouinard *et al.* [30]. Briefly, 40 µl of methyl acetate was added to the phospholipid sample followed by vortexing. Then 40 µl of methylation reagent (0.9 ml of 30% sodium methoxide in 4.1 ml methanol; Fisher Scientific Ltd. Loughborough, UK) was added to each reaction. The mixture was briefly vortexed and allowed to react for 10 minutes at room temperature and then 60 µl of termination reagent (0.2 g oxalic acid in 6 ml diethyl ether) was added, followed by brief vortexing. 200 mg of calcium was added and the mixture allowed to stand for 1 hour to absorb the moisture. The samples were centrifuged for 5 minutes at 3400× g and the supernatant transferred to a gas chromatography vial and used directly for gas chromatography. The fatty acid methyl esters were then injected (split ratio 50∶1) into a gas chromatograph (GC 6890; Agilent technologies Ltd, Stockport, UK). Separation of fatty acid methyl esters was performed with a Varian CP-Sil 88 (Crawford Scientific™ Ltd., Strathaven, UK) capillary column with hydrogen as carrier gas. Oven temperature was programmed from 59°C to 100°C at 8°C per min, then to 170°C at 6°C per minute and held for 10 minutes, and then to 240°C at 3°C per min and held for 10 min. The temperature of the injector and detector were set at 255°C and 250°C respectively. The fatty acid methyl esters were identified by comparing the retention times with a fatty acid methyl esters standard mixture (Sigma-Aldrich Co LLC, Gillingham, UK) and the area percentage in moles were used for the statistical analysis. A total of 36 fatty acids were analyzed in this study and included: saturated fatty acids: C6:0, C8:0, C10:0, C11:0, C12:0, C14:0, C15:0, C16:0, C17:0, C18:0, C20:0, C21:0, C22:0, C23:0, C24:0; monounsaturated fatty acids: C14:1, C15:1, C16:1, C17:1, C20:1, C24:1, and polyunsaturated fatty acids: omega (n)-3: C18:3n3, C20:3n3, C20:5n3, C22:6n3, omega (n)-6: C18:2n6t, C18:2n6, C20:3n6, C20:4n6, and omega (n)-9: C18:1n9t, C18:1n9C, C20:1n9.

#### Leptin protein sequence analysis, alignments, molecular modeling and relationship tree

Swiss-Model (swiss.model.expasy.org) [31] was used to generate a molecular model of the derived African elephant leptin protein sequence (Figure S1) based on the X-ray crystal structure of the human leptin protein (1AX8.pdb) [32] essentially as previously described [33]. The Swiss-PDB viewer scenes were rendered with PovRay (www.povray.org). A previous phylogeny reported for the class I helical cytokines of which leptin is a member did not include elephant [34]. As the class I helical cytokine family is diverse and cannot be confidently compared in a single alignment we generated separate alignments for each family and generated phylogenies using Phyml (WAG model and 100 replicates were used to generate bootstrap values). These phylogenies were then combined into a single tree by hand and colored by family. Figures were generated with *Figtree* (http://tree.bio.ed.ac.uk/software/figtree/).

### Immunohistochemistry

Formalin-fixed paraffin sections of elephant adrenal glands with surrounding adipose capsules (*n* = 9) were deparaffinized with Histo-Clear II (National Diagnostics, Hessle, UK) and rehydrated through a graded series of ethanol and a final wash in distilled water. Antigen retrieval was achieved using microwave heating twice for 5 minutes in 10 mM Sodium citrate buffer (pH 6.0). The slides were left to cool for 20 minutes before being washed three times in distilled water. Endogenous peroxidase activity was blocked by incubating sections with 3% hydrogen peroxide in distilled water for 30 minutes at room temperature. Sections were rinsed three times in distilled water followed by a 5 minute rinse in PBS. Sections were subsequently incubated with normal horse serum (10 µl/ml; Vectastain Universal Elite ABC kit, Vector Laboratories Burlingame, CA) diluted in PBS and 0.1% Tween-20 (PBST) for 30 minutes followed by incubation with rabbit polyclonal antibodies to Leptin (4 µg/ml; Santa Cruz Biotechnology, Inc., Santa Cruz, USA) diluted in PBST for one hour at room temperature. The sections were rinsed twice in PBS for 5 minutes followed by 30 minutes incubation with biotinylated universal secondary antibodies (20 µg/ml; Vector Laboratories, Burlingame, CA) diluted in blocking buffer. Following two rinses in PBS, sections were labelled with avidin-biotin horseradish complexes using the Vectastain ABC Kit (Vector Laboratories) according to the manufacturer's instructions. After three rinses in PBS, antibody binding was visualized by incubating the sections in 3,3′-diaminobenzidine (DAB Kit, Vector Laboratories) for 2–3 minutes. The sections were briefly washed with tap water, counterstained with hematoxylin and rinsed in running tap water. The sections were dehydrated through graded series of ethanol to xylene and the slides were coverslipped using DPX mounting media (Sigma, St. Louis, MO, USA). The sections were viewed and analyzed for leptin immunoreactivity using a Leica DM5000 B microscope equipped with a Leica DFC350 FX camera.

### Statistical analyses

Data obtained from the AT lipid composition was filtered of poorly measured fatty acids (defined as fatty acids with percentiles below 0.01%) and evaluated using IBM SPSS Statistics 21.0 (IBM, USA). Differences between pregnancy status and AT depot were investigated using the Mann-Whitney test; the results are given as mean ±SEM.

## Results

### Microarray analysis of gene expression in elephant adipose tissue

**Figure 1:**
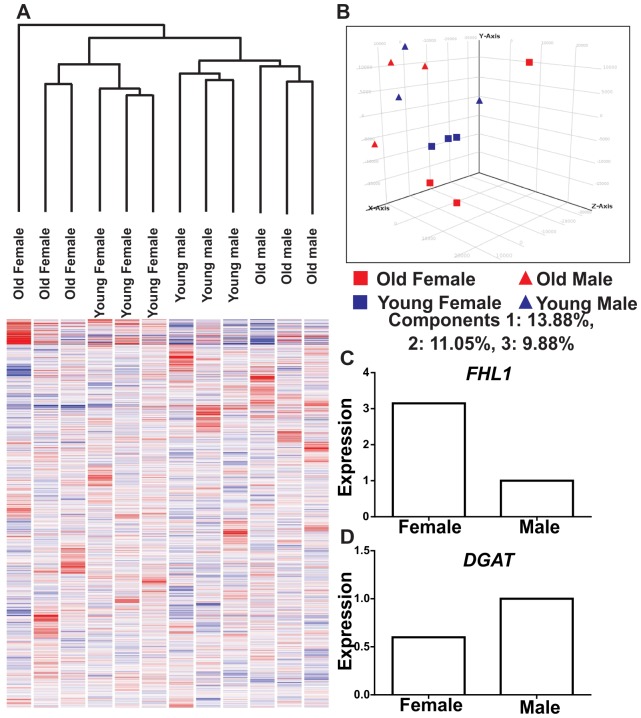
Affymetrix microarray analysis identifies differentially expressed genes in male vs. female and old vs. young African elephant visceral adipose tissue. Hierarchical clustering (A) and principal component analysis (B) shows that samples cluster according to age and sex. Two differentially expressed genes identified by array analysis (A), *DGAT* and *FHL1* were selected for validation by qRTPCR in male (N = 3) and female (N = 3) elephants.

Because the physiology of elephant adipose tissue remains poorly understood, microarray analysis was used to compare the mRNA expression profiles of adipose specimens from young and old, male and female elephants. Cross-species microarray hybridization and analysis approaches are now well developed and permit the study of gene expression in species for which microarray technology is not available [35]. To this end, the perfect match probes from Affymetrix (HT HG_133+PM) oligonucleotide microarrays were used to quantify mRNA expression in RNA from elephant adipose tissue. mRNA expression was compared in adipose tissue from young (<4 years old) and post-pubertal, older (>15 years) elephants, and between male and female elephants. The filtering and statistical parameters outlined in the methods section were utilized, and 20 genes were found to be significantly differentially expressed between the male and female samples and 7 genes found to be significantly differentially expressed between the old and young samples in either sex (Table 1). Hierarchical clustering and principal component analysis distinguished gene expression patterns by age and sex (Figure 1A, B). The NCBI-Database for annotation, visualization and integrated discovery (DAVID) [36] was used to analyze and annotate the gene list identified by microarray analysis. For the genes identified as being differentially expressed between young and old elephants, gene ontology [37] terms for adipose tissue (GO-TERM_BP_FAT, GO-TERM_CC_FAT, GO-TERM_MF_FAT) were enriched. Because of the limited number of genes identified, cluster analysis was not feasible with the dataset. To validate the microarray analysis two differentially expressed genes (Table 1, 2), *DGAT* and *FHL1* were selected for validation by qRTPCR in male (N = 3) and female (N = 3) elephants. Consistent with the array data, expression of *FHL1* was higher in females, whereas *DGAT* was higher in males.

**Table 2:** Differentially expressed genes identified in old versus young elephant adipose tissues.

Affymetrix ID	Fold Change	Regulation	Official Symbol	Function
201235_PM_s_at	3.0314367	up	BTG2	Negative regulation of cell proliferation, response to peptide hormone stimulus
209189_PM_at	2.7250335	up	FOS	Transcription regulator, aging, cellular response to hormone
200664_PM_s_at	2.5327153	up	DNAJB1	Chaperone mediated protein folding
227099_PM_s_at	2.4746747	up	C11orf96	Uncharacterized protein
200033_PM_at	2.3569188	up	DDX5	Transcription co-regulator,
202704_PM_at	2.0977397	up	TOB1	Transcription corepressor & negative regulation of cell proliferation
201417_PM_at	2.0376813	up	SOX4	Transcription regulator & embryo development

#### Tissue phospholipid composition

**Figure 2:**
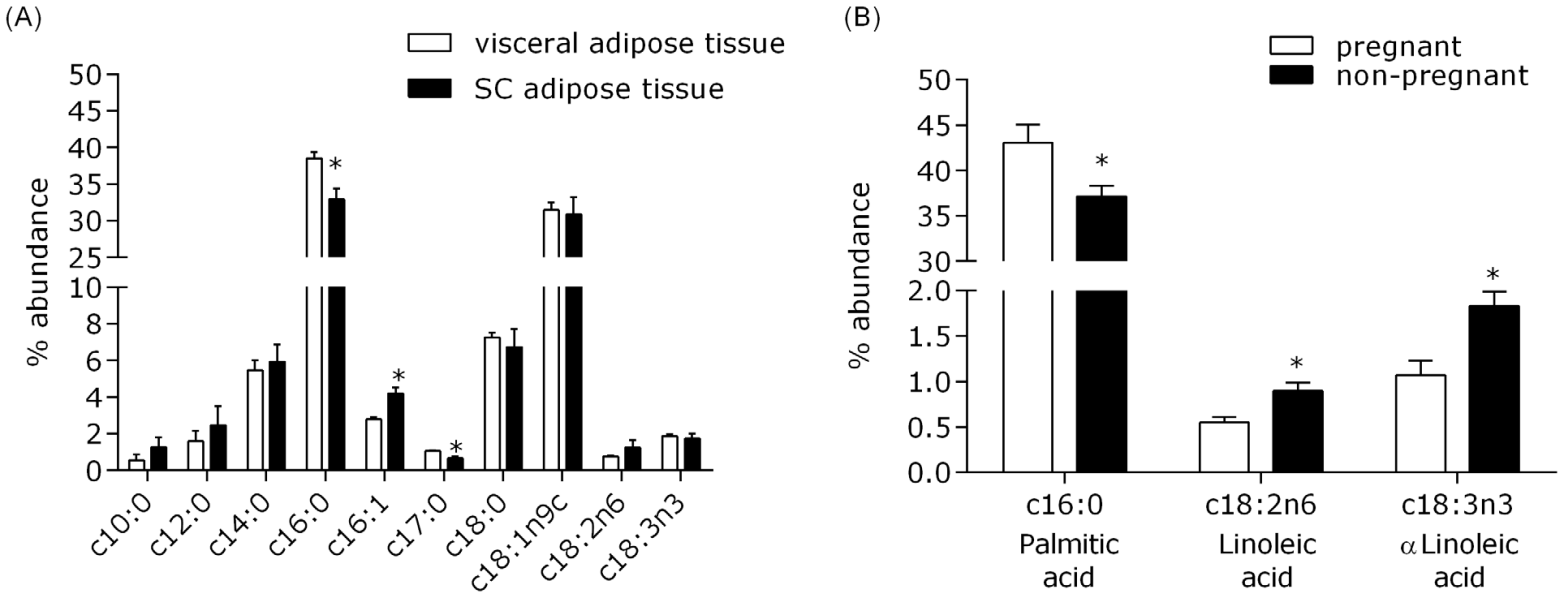
(A) Fatty acid profiles from visceral and subcutaneous adipose tissue. (B) Impact of pregnancy on fatty acid profile in visceral adipose tissue.

Thirty-six fatty acids were identified in African elephant subcutaneous (SC) and visceral (VIS) adipose tissue, although only those with a percentage greater than 0.1 were considered within the analyses (Figure 2). Within both SC and VIS adipose tissue, myristic (C14:0), palmitic (C16:0), stearic (C17:0) and oleic acid (C18:1n9c) displayed the greatest abundance (Figure 2A). Palmitic and heptadecanoic acid (c17:0) were significantly more abundant in VIS adipose tissue whereas palmitoleic (C16:1) was greater in SC adipose tissue (P<0.05). A weak positive relationship was observed between visceral abundance of palmitic acid (the most abundant fatty acid) and age (R^2^ = 0.17, P = 0.038). In accordance with a previous study [38], negative relationships between age and VIS lauric acid (R^2^ = 0.21, P = 0.022), VIS capric acid (R^2^ = 0.18, P = 0.018) and SC lauric acid (R^2^ = 0.67, P = 0.028) were observed. Additionally, when only the female animals were considered, pregnancy was associated with increased palmitic and decreased linoleic and α linoleic acid abundance in VIS adipose tissue (Figure 2B).

### Expression and characterization of leptin mRNA and protein in African elephants

**Figure 3:**
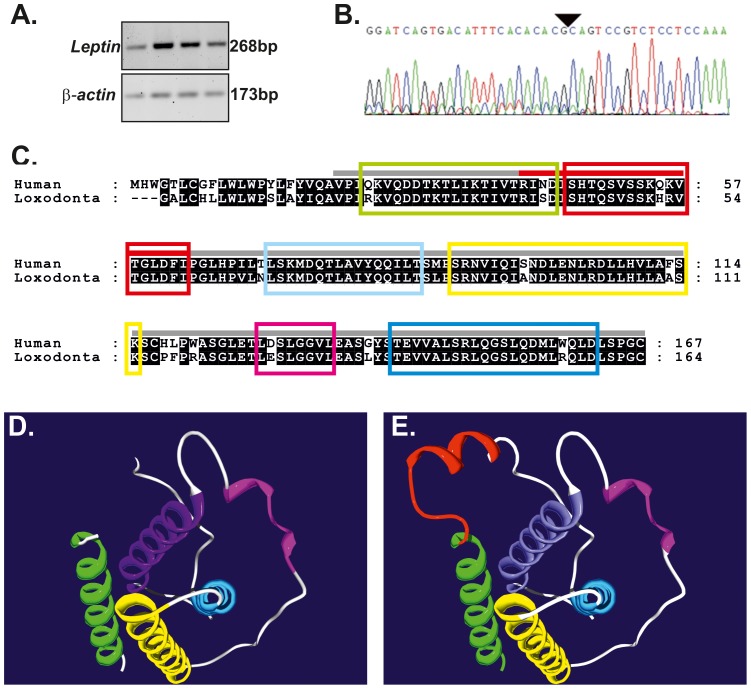
Leptin expression in African elephant visceral fat and comparison of African elephant leptin with the sequence and structure of human leptin. RT-PCR confirms expression of leptin in elephant visceral fat (A) and identified an exon-exon boundary (B), derived amino acid sequence (C), structure of human leptin (1AX8) (D) and molecular model of elephant leptin (E). Residues conserved in all human and elephant leptin amino acid sequences are displayed as white text on black. Helices are color coded (C, D,E).

**Figure 4:**
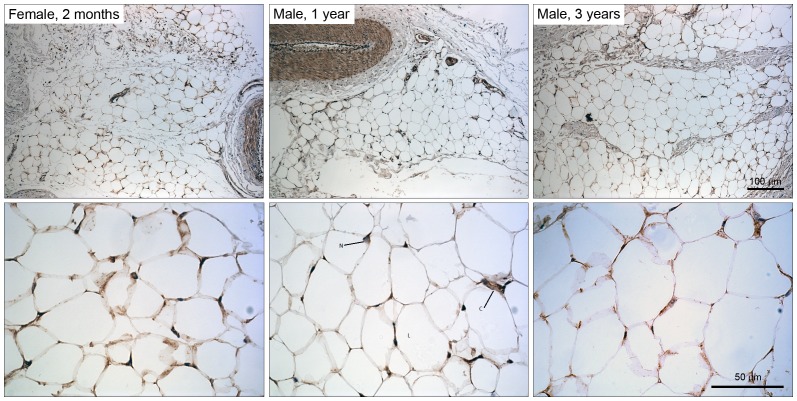
Representative immunohistochemical staining confirming expression of the leptin protein in three independent elephant adipose tissue specimens. Dark brown staining indicates leptin. Leptin was expressed exclusively in the cytoplasm of adipocytes. The top panel shows micrographs of leptin staining at 10× magnification and the lower panel shows micrographs at 40× magnification; N = nucleus, L = lipid droplet, C = cytoplasm.

**Figure 5:**
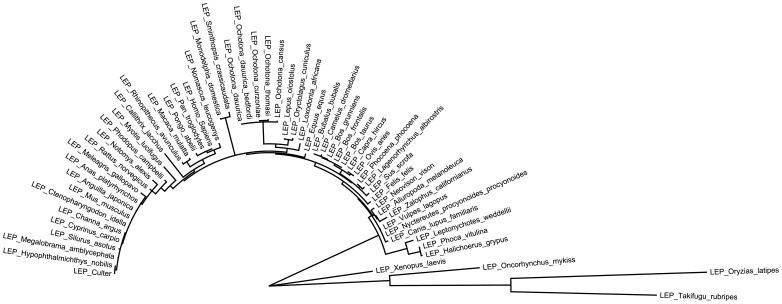
Protein sequence relationship between derived African elephant leptin protein and other members of cyclical cytokine family. The class I helical cytokine superfamily has been described earlier. The sequence relationship between the derived elephant leptin protein sequence and other members of cyclical cytokine family was therefore analyzed. An alignment of class I helical cytokine family members was prepared using MUSCLE and phyml used to generate a tree using the WAG model and 100 replicates were used to generate bootstrap values. These were then combined into a single tree by hand and colored by cytokine sub-family. The relationship of the African elephant leptin protein was determined relative to the sequence in other mammalian species, to contextualize the relationship of elephant leptin structure to that of leptin in other species. The global tree is available as a supplemental figure (Figure S2).

RT-PCR was used to detect expression of *leptin* mRNA in African elephant AT with *actin* used to confirm cDNA integrity (Figure 3A). Automated Sanger DNA sequencing confirmed the identity of the *leptin* transcript, which demonstrated ∼90% similarity with the respective human homologue (NM_000230.2) and confirmed the presence of a conserved exon boundary (Figure 3B). The derived amino acid sequence of the elephant leptin protein was utilized to generate a homology structural model (Figure 3C, D) based on the X-ray crystal structure of the human leptin protein (1AX8) [32]. Given the high degree of identity between the human and elephant leptin protein sequences (∼84%) it seemed reasonable to expect that antibodies against human leptin might also detect elephant leptin. Therefore established immunohistochemical approaches were used to detect the expression and tissue distribution of the leptin protein in elephant AT. This confirmed expression of leptin at the protein level in African elephant adipose tissue (Figure 4). Finally, to further confirm the identity of the putative elephant leptin sequence, the relationship of derived elephant leptin protein sequence (Figure 3C) to the class I helical superfamily was determined. The derived elephant leptin protein sequence is most closely related to the leptin homologs of *Oryctolagus cuniculus* (European rabbit), *Lepus oiostolus* (woolly hare), and members of the *Ochotonidae* (Pika) (Figure 5, Figure S2) within the leptin sub-family of class I helical cytokines.

## Discussion

Since the discovery of leptin, adipose tissue was confirmed as a dynamic and physiologically flexible organ with emerging diverse roles in reproduction, energy sensing and regulation, and inflammatory responses. The physiology of AT and presence of leptin has been investigated in many mammalian species, establishing leptin as a key facilitator of AT functions, particularly by integrating nutritional status and reproduction. To date no one has established the presence of leptin, nor examined fatty acid profiles or adipocyte gene expression in the African elephant. This report provides the first comprehensive characterization of adipose tissue from the African elephant. This molecular knowledge will support the development of novel nutritional and behavioral management strategies to ensure the survival and sustainability of captive elephants.

### Microarray analysis of gene expression

Microarray analysis (Figure 1) suggests elephant adipose tissues share many features with adipose tissue from well-characterized species, including humans and mice (Figure 1). Differentially expressed genes identified between young and old, and male and female elephants (Table 1, 2), were found to have similar functions in humans and mice. Sex related differences in gene expression were first considered (Table 1). To contextualize our findings we have investigated the function of the differentially expressed genes identified here in other biological contexts in the literature. *DGAT2* (diacylglycerol O-acyltransferase 2), a gene responsible for lipid metabolism, as well as fatty acid and triglyceride homeostasis, showed the greatest fold change difference in expression between male and female elephants; it was more than fourfold higher in males, and this finding of differential expression based on sex of the elephant was supported by confirmatory qRTPCR testing (Figure 1C,D). Many studies have reported that levels of plasma leptin are significantly higher in females, both in human and in rats, compared to males, when equated for adiposity and body weight [39]–[41]. Results from the current study are in agreement with this difference; *leptin* expression in female elephants was slightly higher than in males. Suzuki and colleagues [42] reported that expression of *DGAT2* in WAT was downregulated by leptin. Similarly, upregulation of *DGAT2* was demonstrated in *leptin* deficient ob/ob mice [43], whereas intracerebroventricular infusion of leptin resulted in a reduction in DGAT2 expression in WAT [42]. However, some studies report contrasting findings and might suggest that females have higher expression of DGAT2 than males. For example, a decrease in DGAT2 expression was observed in cynomolgus monkeys treated with 5α-dihydrotestosterone (DHT) [44] and in ovariectomized rats [45]. Both of these findings would suggest that under certain conditions, androgens can decrease expression of *DGAT2*, leading to an expectation that females would have higher expression of *DGAT2*. However in the current study, higher *DGAT2* expression was noted in male as compared to female elephant adipose tissue (Figure 1D).

The *ATP5L* gene encodes the mitochondrial ATP synthase subunit g which participates in oxidative phosphorylation ATP synthesis. The higher expression of *ATP5L* in male elephants compared to female (Table 1) may be attributable to the marked sexual dimorphism in size between male and female elephants, with males (average weight 4,100–5,000 kg; average height 2.7–3.2 m at the shoulder) being significantly larger than females (average weight 2,300–4,000 kg; average height 2.3–2.7 m at the shoulder) [46], and consequent differences likely in energy expenditure. Such differences have been demonstrated in other species. Sex differences in energy metabolism in humans were found in both children and adults with adjustment of body composition; indeed, a number of age-matched studies have suggested that whole body energy expenditure is lower in females compared to males [47]. Similarly, in a species of mole-rat, *Bathyergus janetta*, which has sexual dimorphism in size, males have a higher energy expenditure, most likely due to cost for maintenance of their larger body size [48].

The *COL3A1* gene, which encodes the pro-α1 chains of type III collagen and is expressed in adipocytes [49], was also elevated in males compared to females (Table 1). This is consistent with a report from an examination of sex-specific pathways in early cardiac response to pressure overload in mice [50]. Expression of collagen types appears to alter during changes in adiposity [49], [51], therefore it is interesting to note the sex-related differential expression of *COL3A1* in elephants. The *actin* γ2 (*ACTG2*) and β-*actin* (*ACTB*) genes were both higher in females compared to males. This is consistent with a report that mRNA levels for sarcomeric *actin* in the heart of female rats were greater by 79% (P<0.001), and levels of cytoskeletal *actin* were 130% greater in females (P<0.01) compared with levels in age-matched male heart tissue [52].

*PTP4A2*, also known as *PRL2*, is a protein tyrosine phosphatase, expression of which was higher in female elephant adipose tissue compared to males (Table 1). Interestingly, PTP4A2/PRL2 was previously shown to promote signaling by the EPO and IL3 members of the class I cyclical helical family of cytokines [53]. Therefore it is possible that PTP4A2/PRL2 has a similar role in regulating leptin receptor signaling in adipose tissue [54]. Furthermore PTP4A2/PRL2 expression has been linked to signaling by the sex hormones estrogen and progesterone in breast cancer. Microarray and qPCR expression of PTP4A2 expression was elevated in both estrogen receptor (ER)-positive and progestin receptor (PR)-positive breast cancer biopsies compared to ER-negative or PR-negative biopsies [55]. This suggests a link between this gene and female sex hormones, which may in part explain the higher expression of PTP4A2/PRL2 in female elephants.

NR2F2, also known as COUP-TF2, is member of the nuclear receptor superfamily of ligand dependent transcription factors [56]. Mice lacking *NR2F2* exhibit reduced adiposity and NR2F2 has been shown to be a crucial regulator of energy homeostasis and adipogenesis [56]. Consistent with this, ectopic over-expression of *NR2F2* promoted lipid accumulation and adiposity [56]. Thus the higher expression if *NR2F2* in female elephants may be associated with sex-related differences in adipose deposition in elephants (Table 1). Further studies are therefore warranted to ascertain the physiological effects of the identified difference in *NR2F2* expression in male and female elephants and whether this contributes to the impaired reproductive phenotype identified in captive female elephants.

Age related differences in expression of known adipose genes were also noted in the present study; all identified genes were more highly expressed in the sexually mature animals (Table 2). Interestingly, this included a number of genes associated with cellular proliferation. BTG2 controls cell cycle progression and proneural expression genes by acting as a transcription coregulator, and has been implicated in intra-muscular fat deposition in beef cattle, potentially by influencing pre-adipocyte cellular differentiation [57]. The Fos protein heterodimerizes with Jun to form the AP1 transcription factor complex, and regulates cell proliferation, differentiation and transformation, and has long been implicated in adipose response to insulin [58] and preadipocyte differentiation [59]. The DDX5 RNA helicase, which like Fos was elevated in older elephants, has a role in promoting estrogen-activated Fos expression [60]. DDX5, also referred to as p68, promotes androgen and estrogen receptor transcriptional activation functions [61]. Thus, increased DDX5 expression in older elephant AT may enhance the tissues' hormonal responsiveness and thereby alter adipose metabolism.

### Adipose tissue fatty acid profile

This is the first report of the fatty acid profile of both VIS and SC adipose tissue from the African elephant. In accordance with a previous study by Duncan and Garton [38], palmitic acid was the most abundant fatty acid in VIS adipose tissue; results from the current study confirmed that this is also the case in SC adipose tissue. This is in contrast to other herbivore species, where c18:1 (oleic acid) is usually the most abundant fatty acid [62], [63]. Interestingly, heptadecanoic acid (C17:0) was present within both VIS and SC adipose tissue; this fatty acid is generated by bacterial degradation in ruminant species and is therefore only usually present in ruminant tissue and milk and acts a specific marker of intake of these products in humans [64], [65]. However, as a hindgut fermenter, the elephant also appears to be able to generate these fatty acid species. The negative relationship between VIS capric, SC and VIS lauric acid and age is in accord with the findings of Duncan and Garton; these medium chain fatty acids may be derived from maternal milk, as elephant milk, in comparison with other species, is particularly rich in capric acid (65%) and lauric acid (17%) [66]–[68].

An increase in body fat is observed in many species during pregnancy, which supports fetal and mammary tissue growth. Palmitic acid in ester form represents a major storage component of triglycerides and is the first fatty acid produced in de novo fatty acid synthesis. The increase in palmitic acid observed in VIS adipose tissue in the pregnant elephants may be attributed to a normal gestational increase in lipid storage for oxidation postpartum. Although comprising a low abundance within adipose tissue (Figure 2), both linoleic and α linoleic acid are essential fatty acids (EFA) and crucially important for the normal development of the fetus [17], in particular as components of cell membranes within the nervous system [69]. The abundance of EFAs is known to decrease during pregnancy in humans (as assessed in plasma phospholipids) and results from this study confirm that this is also true for elephant VIS adipose tissue [70]. However, the profile of EFAs demonstrating a decline during pregnancy is different; humans experience no change in linoleic acid, but a decrease in docosahexaenoic acid (DHA) [70]. Whether captive species experience a similar decline in EFAs during pregnancy is unknown, but the significance of these compositional differences in adipose tissue fatty acid profiles may be important for the formulation of optimal pregnancy and lactation diets for captive animals.

### Leptin expression, sequence and model

Leptin has long been recognized to be a crucial link between adipose tissue function and reproduction ([71] and references therein). However the elephant leptin gene has remained poorly characterized. To address this, the *leptin* gene was identified within the African elephant genome sequence and reverse transcriptase PCR was used to confirm expression of *leptin* mRNA in elephant adipose tissue (Figure 3A). Sanger sequencing was used to determine the sequence, and results confirmed that *leptin* gene structure is conserved between humans and elephants (Figure 3B). The cDNA derived protein sequence (Figure 3C) was used to generate a structural homology model based on the human leptin crystal structure (1AX8) [32]. Interestingly, amino acids affected by pathogenic mutations identified in human patients exhibiting leptin resistance [72]–[74] were conserved in elephant leptin, suggesting a conservation of leptin function in humans and elephant at the molecular level. Given the high degree of conservation (∼84%) between human and elephant leptin, it seemed reasonable to expect that antibodies raised against the human leptin protein would also detect the elephant ortholog (Figure 3, 5). As a proof of principle, immunohistochemistry was used to detect expression of the leptin protein in the cytoplasm of adipocytes (Figure 4). Future studies are therefore feasible to address the role of leptin in elephant reproductive studies.

## Conclusions

This study provides the first molecular characterization of elephant adipose tissue. Similarities were identified between the genes expressed in adipose tissue from elephants and several other species. The fatty acid profiles from adipose tissue depots of free ranging elephants will provide useful information for dietary modifications of captive animals. Future studies are therefore warranted to address the potential relationship between body condition score, leptin function and reproductive impairment in elephants.

## Supporting Information

Figure S1 **Derived amino acid sequence of the leptin protein from the African elephant.**
(PDF)

Figure S2 **Global tree class II helical cytokine tree.** Sequences used were as follows (LEPTIN: NP_001180601.1, NP_001036220.1, NP_001009850.1, NP_001157452.1, NP_001139362.1, NP_001089183.1, NP_000221.1, NP_032519.1, NP_037208.1, NP_001027897.1, NP_001098190.2, NP_001156541.1, XP_003261338.1, XP_002818456.1, XP_004008087.1, AAT45394.1, ABV79899.1, ACV13199.1, CAJ43200.2, ACE87887.1, XP_002913466.1, NP_776353.2, NP_999005.1, ACF40216.1, ACD85081.1, ADZ16385.1, NP_001003070.1, AAT45398.1, ABG81864.1, ABL74887.1, AAT28186.1, Q706D0.1, CAI99387.1, Q9XSW9.1, Q706D1.1, ABK88255.1, XP_001366398.1, ABL74883.1, ABL74885.1, ABB90403.1, ABN13964.1, ABL74882.1, O93416.1, XP_002752099.1, CAJ43198.1, AAT45396.1, AAT38807.1, AAT37636.1, AAT45399.1, AAT45397.1, AAO91910.1, CAJ43201.1, AAO19891.1, AAM21764.1, AAM21763.1, AAL16404.1. EPO: NP_001075294.1, NP_031968.1, NP_058697.1, NP_001184210.1, NP_001108600.1, NP_001108600.1, NP_001033098.1, NP_000790.2, NP_001006647.1, NP_001075559.1, NP_001019908.1, NP_001036201.1, NP_776334.1. GH: NP_001018328.2, NP_001009315.2, NP_000506.2, NP_032143.1, NP_001187174.1, NP_001030020.2, NP_851339.1, NP_001003168.1, NP_989690.1, NP_999034.2, NP_001036203.1, NP_001184093.1, NP_001009337.1, NP_001166330.1, NP_001075417.1, NP_001117148.1, NP_001028165.1, NP_001076144.1. PRL: NP_001156326.1, NP_852102.2, NP_000939.1, NP_001093699.1, NP_001028166.1, NP_001159915.1, NP_990797.2, NP_001182038.1, NP_001038167.1, NP_036761.1, NP_064464.1, NP_001037736.1, NP_001077409.2, NP_001072092.1, NP_001040593.1, NP_001009306.1, NP_001157002.1, NP_036084.2, NP_714958.1, NP_082753.1, NP_080172.1, NP_776378.2, NP_001075365.1, NP_999091.1, NP_001008276.2, NP_001118205.1, NP_001117140.1, NP_001036806.1, NP_034218.1, NP_862900.1, NP_742168.1. IL6: NP_000591.1, NP_036721.1, NP_112445.1, NP_989959.1, NP_001003301.1, NP_001118129.1, NP_999564.1, NP_776348.1, NP_001075965.1, NP_001075533.1, NP_001036198.2, NP_001009211.1, NP_001009392.1, NP_001167007.1. GCSF: NP_000750.1, NP_001075329.1, NP_058800.1, NP_034101.1, NP_776453.1, NP_999007.1, NP_001009227.1. LIF: NP_999567.1, NP_002300.1, NP_001184002.1, NP_032527.1, NP_071532.2, NP_776356.1. OSM: NP_065391.1, NP_001181403.1, NP_783644.3, NP_001006962.1, NP_001013383.1. CNF: NP_740756.1, NP_037298.1, NP_000605.1, NP_990823.1, NP_001075752.1. CCF1: NP_001159684.1, NP_037378.1, NP_064336.1, NP_001016067.1, NP_997498.2. CT1: NP_001321.1, NP_001136016.1, NP_031821.1, NP_058825.1, NP_001179313.1, NP_001129272.1, NP_942155.1, NP_001009112.1).
(PDF)
