# Supplementary material for: Molecular Characterization of Adipose Tissue in the African Elephant (Loxodonta africana)
Source: PLoS One. 2014 Mar 14;9(3):e91717. doi: 10.1371/journal.pone.0091717 (PMC3954733; doi:10.1371/journal.pone.0091717)
Supplement: Figure S1 — Derived amino acid sequence of the leptin protein from the African elephant. (PDF) [file pone.0091717.s001.pdf]

Supplemental Figure 1

```
>LEP_LOXODONTA AFRICANA
GALCHLLWLWPSLAYIQAVPIRKVQDDTKTLIKTIVTRISDISHTQSVSSKHRVTGLDFIPG
LHPVLNLSKMDQTLAIYQQILTSLPSRNVIQIANDLENLRDLLHLLAASKSCPFPRASGLET
LESLGGVLEASLYSTEVVALSRLQGSLQDMLRQLDLSPGC
```
