# Supplementary figures and images for: Molecular Characterization of Adipose Tissue in the African Elephant (Loxodonta africana)
Source: PLoS One. 2014 Mar 14;9(3):e91717. doi: 10.1371/journal.pone.0091717 (PMC3954733; doi:10.1371/journal.pone.0091717)

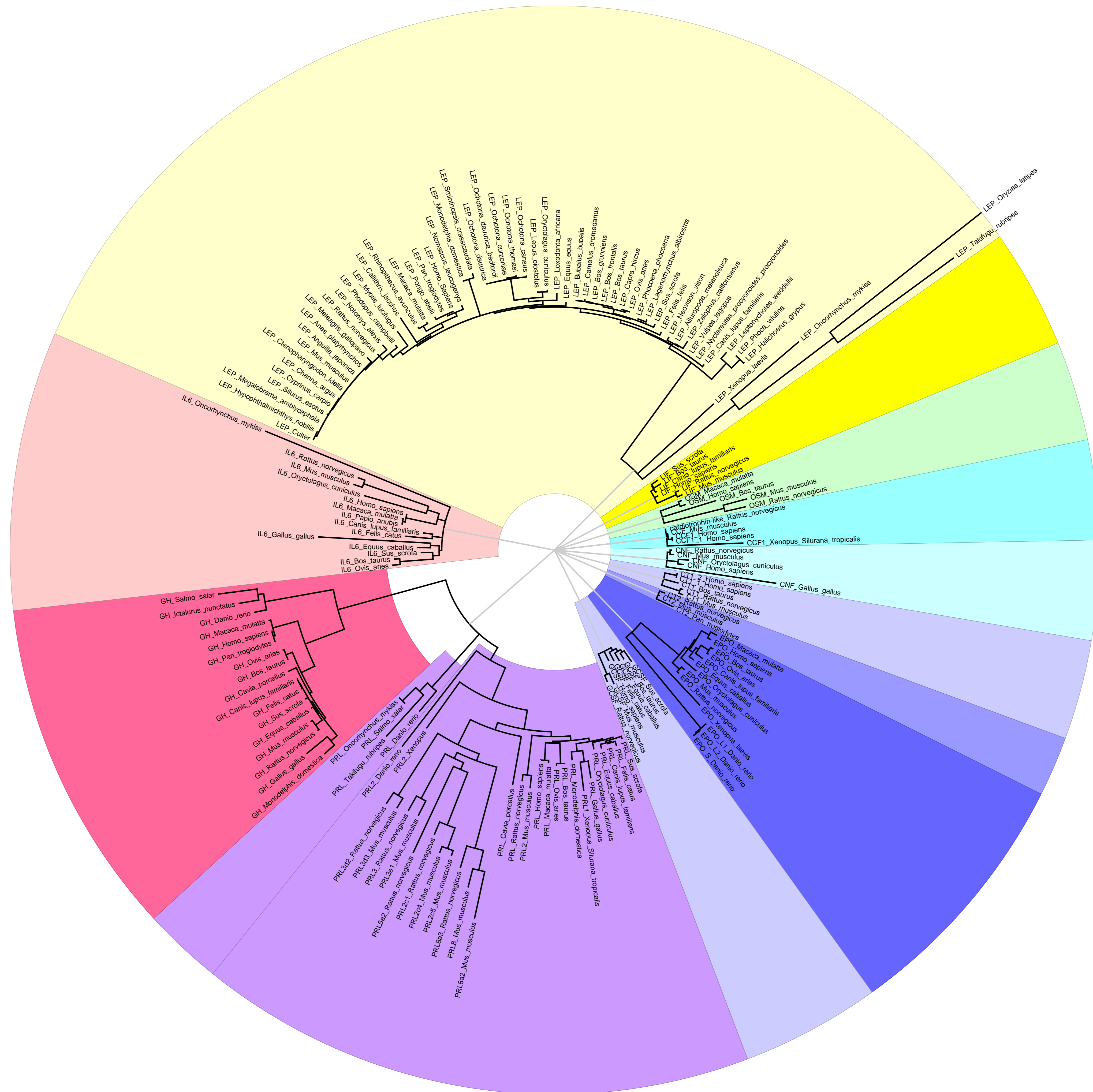

Supplement: Figure S2 — Global tree class II helical cytokine tree. Sequences used were as follows (LEPTIN: NP_001180601.1, NP_001036220.1, NP_001009850.1, NP_001157452.1, NP_001139362.1, NP_001089183.1, NP_000221.1, NP_032519.1, NP_037208.1, NP_001027897.1, NP_001098190.2, NP_001156541.1, XP_003261338.1, XP_002818456.1, XP_004008087.1, AAT45394.1, ABV79899.1, ACV13199.1, CAJ43200.2, ACE87887.1, XP_002913466.1, NP_776353.2, NP_999005.1, ACF40216.1, ACD85081.1, ADZ16385.1, NP_001003070.1, AAT45398.1, ABG81864.1, ABL74887.1, AAT28186.1, Q706D0.1, CAI99387.1, Q9XSW9.1, Q706D1.1, ABK88255.1, XP_001366398.1, ABL74883.1, ABL74885.1, ABB90403.1, ABN13964.1, ABL74882.1, O93416.1, XP_002752099.1, CAJ43198.1, AAT45396.1, AAT38807.1, AAT37636.1, AAT45399.1, AAT45397.1, AAO91910.1, CAJ43201.1, AAO19891.1, AAM21764.1, AAM21763.1, AAL16404.1. EPO: NP_001075294.1, NP_031968.1, NP_058697.1, NP_001184210.1, NP_001108600.1, NP_001108600.1, NP_001033098.1, NP_000790.2, NP_001006647.1, NP_001075559.1, NP_001019908.1, NP_001036201.1, NP_776334.1. GH: NP_001018328.2, NP_001009315.2, NP_000506.2, NP_032143.1, NP_001187174.1, NP_001030020.2, NP_851339.1, NP_001003168.1, NP_989690.1, NP_999034.2, NP_001036203.1, NP_001184093.1, NP_001009337.1, NP_001166330.1, NP_001075417.1, NP_001117148.1, NP_001028165.1, NP_001076144.1. PRL: NP_001156326.1, NP_852102.2, NP_000939.1, NP_001093699.1, NP_001028166.1, NP_001159915.1, NP_990797.2, NP_001182038.1, NP_001038167.1, NP_036761.1, NP_064464.1, NP_001037736.1, NP_001077409.2, NP_001072092.1, NP_001040593.1, NP_001009306.1, NP_001157002.1, NP_036084.2, NP_714958.1, NP_082753.1, NP_080172.1, NP_776378.2, NP_001075365.1, NP_999091.1, NP_001008276.2, NP_001118205.1, NP_001117140.1, NP_001036806.1, NP_034218.1, NP_862900.1, NP_742168.1. IL6: NP_000591.1, NP_036721.1, NP_112445.1, NP_989959.1, NP_001003301.1, NP_001118129.1, NP_999564.1, NP_776348.1, NP_001075965.1, NP_001075533.1, NP_001036198.2, NP_001009211.1, NP_001009392.1, NP_001167007.1. GCSF: NP_000750.1, NP_001075329.1, [file pone.0091717.s002.pdf]
